# Evaluation of a Decision Support System Developed with Deep Learning Approach for Detecting Dental Caries with Cone-Beam Computed Tomography Imaging

**DOI:** 10.3390/diagnostics13223471

**Published:** 2023-11-18

**Authors:** Hakan Amasya, Mustafa Alkhader, Gözde Serindere, Karolina Futyma-Gąbka, Ceren Aktuna Belgin, Maxim Gusarev, Matvey Ezhov, Ingrid Różyło-Kalinowska, Merve Önder, Alex Sanders, Andre Luiz Ferreira Costa, Sérgio Lúcio Pereira de Castro Lopes, Kaan Orhan

**Affiliations:** 1 Department of Oral and Maxillofacial Radiology, Faculty of Dentistry, Istanbul University-Cerrahpaşa, Istanbul 34320, Türkiye; h-amasya@hotmail.com; 2 CAST (Cerrahpasa Research, Simulation and Design Laboratory), Istanbul University-Cerrahpaşa, Istanbul 34320, Türkiye; 3 Health Biotechnology Joint Research and Application Center of Excellence, Istanbul 34220, Türkiye; 4 Department of Oral Medicine and Oral Surgery, Faculty of Dentistry, Jordan University of Science and Technology, Irbid 22110, Jordan; mmalkhader@just.edu.jo; 5 Department of Oral and Maxillofacial Radiology, Faculty of Dentistry, Mustafa Kemal University, Hatay 31060, Türkiye; dt.gozde@hotmail.com (G.S.); dtcaktuna@gmail.com (C.A.B.); 6 Department of Dental and Maxillofacial Radiodiagnostics, Medical University of Lublin, 20-093 Lublin, Poland; lek.dent.karolina.futyma@gmail.com (K.F.-G.); or rozylo.kalinowska@umlub.pl (I.R.-K.); 7 Diagnocat, Inc., San Francisco, CA 94102, USA; m.gusarev@diagnocat.com (M.G.); matvey@diagnocat.com (M.E.); alex@diagnocat.com (A.S.); 8 Department of Oral and Maxillofacial Radiology, Faculty of Dentistry, Ankara University, Ankara 0600, Türkiye; merveonder_16@hotmail.com; 9 Postgraduate Program in Dentistry, Cruzeiro do Sul University (UNICSUL), São Paulo 08060-070, SP, Brazil; alfcosta@gmail.com; 10 Science and Technology Institute, Department of Diagnosis and Surgery, São Paulo State University (UNESP), São José dos Campos 01049-010, SP, Brazil; sergio.lopes@unesp.br; 11 Research Center (MEDITAM), Ankara University Medical Design Application, Ankara 06560, Türkiye; 12 Department of Oral Diagnostics, Faculty of Dentistry, Semmelweis University, 1088 Budapest, Hungary

**Keywords:** dental caries, cone-beam computed tomography, machine learning, decision support systems

## Abstract

This study aims to investigate the effect of using an artificial intelligence (AI) system (Diagnocat, Inc., San Francisco, CA, USA) for caries detection by comparing cone-beam computed tomography (CBCT) evaluation results with and without the software. 500 CBCT volumes are scored by three dentomaxillofacial radiologists for the presence of caries separately on a five-point confidence scale without and with the aid of the AI system. After visual evaluation, the deep convolutional neural network (CNN) model generated a radiological report and observers scored again using AI interface. The ground truth was determined by a hybrid approach. Intra- and inter-observer agreements are evaluated with sensitivity, specificity, accuracy, and kappa statistics. A total of 6008 surfaces are determined as ‘presence of caries’ and 13,928 surfaces are determined as ‘absence of caries’ for ground truth. The area under the ROC curve of observer 1, 2, and 3 are found to be 0.855/0.920, 0.863/0.917, and 0.747/0.903, respectively (unaided/aided). Fleiss Kappa coefficients are changed from 0.325 to 0.468, and the best accuracy (0.939) is achieved with the aided results. The radiographic evaluations performed with aid of the AI system are found to be more compatible and accurate than unaided evaluations in the detection of dental caries with CBCT images.

## 1. Introduction

Dental caries is a multifactorial chronic disease that causes mineral loss in tooth hard tissues. The disease, which has evidence in fossil samples, has a high prevalence today [1,2,3]. Symptoms such as pain, swelling, and abscess may be seen depending on the stage of the disease [4,5,6,7,8]. Caries diagnosis is a clinical decision regarding the presence of caries, while the detection of caries is a result of the clinical and radiographic evaluation of caries signs. Dental probes used for tactile feedback during visual inspection may damage weakened tooth tissues. Although bitewing radiographs are successful in showing the posterior approximal surfaces, they require attention to the rules of projection geometry in the production of images [1,7,8,9]. Cone-beam computed tomography (CBCT) is a volumetric imaging tool which require less patient dose than the medical CTs but more when compared to other plain imaging methods in dentistry. The SEDENTEXCT Panel in 2011 concluded that evaluating dental caries is not an indication for CBCT. However, dental caries findings in volumetric data taken for other reasons should be evaluated [10,11,12].

Digital radiology provides number-based images, paving the way for the development of a clinical decision support system (CDSS) to be integrated into clinical workflows [13,14,15,16]. Moreover, such systems can be developed using artificial intelligence (AI) techniques on subjects such as acute care management, drug ordering, clinical oncology, and many more [17,18,19,20,21,22,23]. AI tools can be developed using a machine learning approach, mainly based on supervised learning, unsupervised learning, semi-supervised learning, and reinforced learning. In supervised learning, data are labeled by experts, while in unsupervised learning, features are extracted by algorithms. In semi-supervised learning, a combination of these two approaches is applied, while in reinforcement learning, the model is developed by giving reward or punishment according to its outputs [24,25,26]. Deep learning, generally combined with the transfer learning approach, is a popular technique used in the automation of tasks such as lesion detection, segmentation, and classification in radiographic data [27,28,29].

The potential of using CBCT volumes for caries detection has been evaluated in several studies [11,30,31,32]. Some researchers proposed AI models for the detection of dental caries with different imaging modalities such as periapical, panoramic, or bitewings [33,34,35]. Lee et al. developed a convolutional neural network (CNN) for dental caries detection and diagnosis using periapical images. A total of 3000 periapical radiographs were labeled as dental caries and non-caries based on medical records and expert evaluation (equal in numbers), then the images were cropped to show one tooth per image and resized to 299 × 299 pixels. The dataset was split into training and testing subsets with the ratio of 4:1, randomly, and the training dataset was augmented 10 times using rotation, width and height shifting, zooming, shearing, and horizontal flipping. The model was based on a pre-trained GoogLeNet Inception v3 CNN network and trained using transfer learning. The model had 9 inception modules, including an auxiliary classifier, two fully connected layers, and softmax functions. The data were given in batches of 32, and 1000 epochs were run at a learning rate of 0.01; the model was fine-tuned by optimizing the weights. The diagnostic performance of the developed model was reported between 82.0–89.0% according to anatomical regions, while AUC values varied between 845 (95% CI 0.790–0.901) and 0.917 (95% CI 0.860–0.975) [33]. Bui et al. proposed a computer-aided diagnosis (CAD) system to detect dental caries in panoramic radiographs. The dataset consisted of a total of 533 single-tooth images (229 caries and 304 non-caries) which was manually segmented from panoramic radiographs. The system was based on two modules: feature extraction and classification. In the first module, the pre-trained CNN models such as Alexnet, Googlenet, VGG16, VGG19, Resnet18, Resnet50, Resnet101, and Xception were used to extract the deep activated features. The extracted features were optimized using mathematical descriptors, such as mean and STD, and texture features such as Haralick’s features; the results of the deep networks and geometric features were optimized mainly based on a Support Vector Machine (SVM) model, prior to being fed into the second module. The fusion features were tested using SVM, Naïve Bayes, k-nearest neighbor, decision tree, and random forest classifier models, and the authors reported that the proposed method achieved 91.70%, 90.43%, and 92.67% accuracy, sensitivity, and specificity, respectively [34]. Devito et al. proposed an artificial multilayer perceptron neural network for the diagnosis of proximal dental caries using bitewing radiographs. The tooth surfaces were divided as sound and dental caries (non-cavitated) by visual evaluation, and a total of 40 pre-molar and 40 molar-extracted teeth were embedded in silicone models to develop 20 tooth models, including canines for proximal contact. The neural network was based on a one-hidden layer perceptron model with a back-propagation algorithm and had 25 neurons in the input and the hidden layer, and 1 in the output layer. Samples were divided into training, testing and cross-validation subsets with the ratio of 2:1:1, and the initial weights of each “synapse” was determined using the Nguyen–Windrow algorithm. A total number of 160 tooth surfaces were scored by 25 examiners (from 1 to 5), each with over 20 years’ experience, and each result was given as inputs. The training was optimized by analyzing the reduction in the mean square error. The golden standard was obtained by histopathological evaluation of the samples after the radiographic acquisition. The area under the ROC curve was reported to be 0.884, and the developed model performed 23.3% better than the best examiner and 39.4% better than the mean human performance [35]. Cantu et at. developed a fully CNN model based on U-Net architecture to detect caries lesions with varying radiographic stages on bitewing radiographs. A total of 3686 bitewings were labeled by three expert dentists in a pixel-wise fashion, and a fourth expert dentist reviewed and revised the process. Each annotation was further classified into four categories by two independent dentists according to the radiographic stage. All images were resized to 512 × 416 pixels, and the data were divided into a training (3293), validation (252), and test dataset (141). Researchers initialized the current model’s weights using the data obtained in a previously developed model for caries segmentation on panoramic radiographs (unpublished) and then applied data augmentation techniques based on geometric level (image flipping, center cropping, xy-translation, and rotations) and pixel level (gaussian-blur, sharpening, contrast, and brightness) random transformation techniques to improve the generalization of the previous model. Several models were trained with different training strategies, loss functions, and combinations of the parameters to improve the performance. First, they started with 10 epochs with a constant encoder weight and a learning rate value of 5e^−3^. Then, the training was further improved for 190 epochs by allowing the optimization of weights in all layers, with a batch size of two and an initial learning rate of 5e^−4^. The results of each epoch were saved and improvements in the mean Intersection-over-Union (IoU) were analyzed. After adjusting the optimal weights, the outputs were converted into binary results by determining a cutoff threshold using Adam optimizer. Further, the system’s results were compared with a cohort of seven dentists with 3–14 years of experience. The authors reported a higher accuracy (0.80) in results of the model when compared to the dentists (0.71) and significantly more sensitivity when compared to the dentists (0.75 versus 0.36). The specificity of the model (0.83) was found to be lower than the dentists (0.91); however, the results were not significant [36].

This study aims to investigate the effect of using a CDSS (Diagnocat, Inc., San Francisco, CA, USA) with caries detection function to enhance CBCT evaluations in terms of detecting dental caries by comparing the results of the observers with and without the aid of the software. For this purpose, volumetric data that met the criteria such as absence of gross artifacts and presence of sufficient teeth were collected among CBCT volumes obtained for other clinical reasons; this study does not support the justification of the use of CBCT only for caries diagnosis. According to the literature review conducted on Pubmed and Google Scholar using the keywords of “caries” or “dental caries” and “cbct” or “cone beam computed tomography” or “cone-beam computed tomography” and “artificial intelligence” or “AI” or “machine learning” or “ML”, no other study was found on this subject other than the Diagnocat system. Hence, this study contributes to the literature in terms of evaluating the performance of an AI model developed for the evaluation of secondary diagnoses using CBCT images obtained in real clinical conditions.

## 2. Materials and Methods

Using retrospective data from our faculty, a power analysis (Power and Precision software, Biostat, Englewood, NJ, USA) was conducted which indicated that detection of differences between the observers with and without the aid of the software could be obtained with 432 CBCT volumes and at least 1098 caries at a power of 0.8 (alpha = 0.05). Thus, this study conducted 500 CBCT volumes retrospectively and all caries lesions were included in the CBCT volumes of the patients between 18 and 64 years of age selected from a Jordan Technological University Hospital’s database.

Patients with fixed prosthetics, implants, caries lesions, missing, or restored teeth were included, while edentulous patients and volumes with exceeding artifacts were excluded. This study was approved by the Institutional Research Board of Jordan Institute of Technology with the protocol number of 792-2019. Informed consent was obtained from all individual participants included in the study. CBCT volumes of the patients were acquired by CS 8100 3D (Carestream Health,, NY, USA) CBCT machine in a standing position during imaging. The scanner offers multiple fields of view (FOVs), allowing the dentist to select the optimum scan on a case-by-case basis. Digital radiographs were acquired with the imaging parameters of 80 kVp, 6 mA (6300 µA), 15 s of imaging time, and 8 × 9 FOV (0.150 mm^3^ voxel size) with isotropic voxels. All cases were selected from the database to be examined by the decision of a dentomaxillofacial radiology consultant with more than 10 years of experience. The radiographic data were anonymized (except gender and age) and CBCT volumes were exported in DICOM format. The dataset was split into encrypted compressed files and distributed to independent observers for radiographic evaluation using the cloud service.

**Figure 1:**
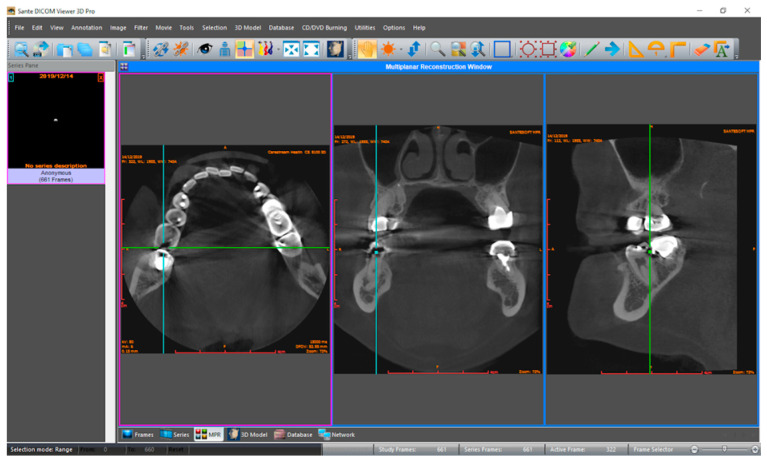
Interface for unaided evaluations in the multiplanar reconstruction view (Sante DICOM Viewer Pro for macOS). In multiplanar (MPR) reconstruction mode, the purple frame indicates the active plane (axial in this case), while the blue frames represent other dimensions which follow the actions in the active plane. The green lines represent the intersection point in all three planes, which demonstrate an approximal dental caries in the distal surface of the tooth number 36. Thus, findings in the active frame are evaluated together with other axes.

**Figure 2:**
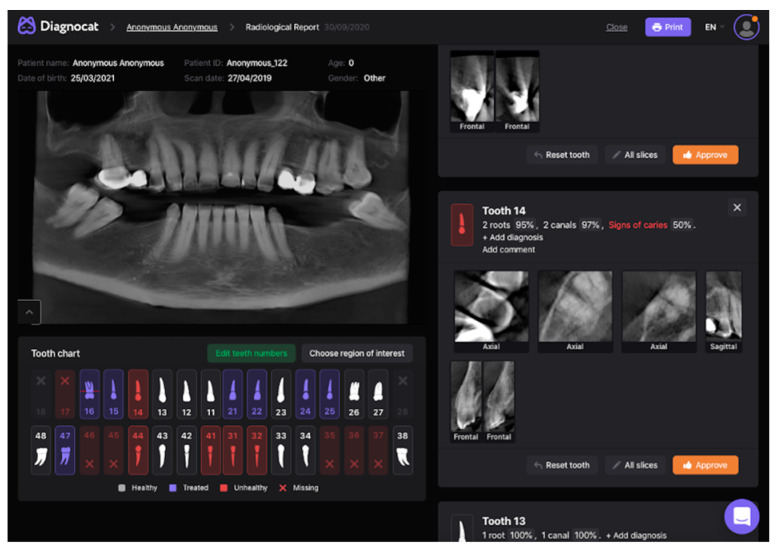
Interface for aided evaluation (Diagnocat). On the left side, at the top, the synthetic–panoramic image produced from the CBCT volume is provided for an overall view, while underneath, there is a dental chart that provides information about the condition of each tooth. The colors of white and purple represent a healthy and treated tooth, while the red means an unhealthy or missing tooth. On the right, the predictions of the system for the relevant tooth are provided by image slices in different axes.

Three observers in dentomaxillofacial radiology evaluated CBCT volumes for dental caries signs, without and with the Diagnocat system. An online conference was conducted prior to evaluations for the calibration of the observers with different levels of experience. The results of aided and unaided evaluations were collected in a template document to ensure standardization among observers. The template with dedicated columns for ‘tooth condition’, ‘mesial surface’, and ‘distal surface’ for each tooth was prepared to collect the responses in an organized manner. Tooth conditions were saved as ‘intact’, ‘missing’, ‘restorated’, ‘support’ and ‘excluded’. Mesial and distal surfaces of the tooth were scored by independent observers separately for the presence of caries on a five-point confidence scale: (1) caries definitely absent, (2) caries probably absent, (3) unsure, (4) caries probably present, and (5) caries definitely present. Primarily, the dataset was imported to Sante DICOM Viewer Pro (Santesoft Ltd., Nicosia, Cyprus) by each observer independently (version 11.6.2 for Windows, 2.0.1 for macOS), and unaided evaluations were performed without any restriction and saved (Figure 1). After a month-long time interval, the dataset was uploaded to the Diagnocat system, and CBCT volumes were analyzed to generate a radiological report. Observers were granted access to the web-based system (Figure 2) to re-evaluate the samples with the aid of the Diagnocat system, and the results were saved using new duplicates of the template.

### 2.1. Model Pipeline

**Figure 3:**
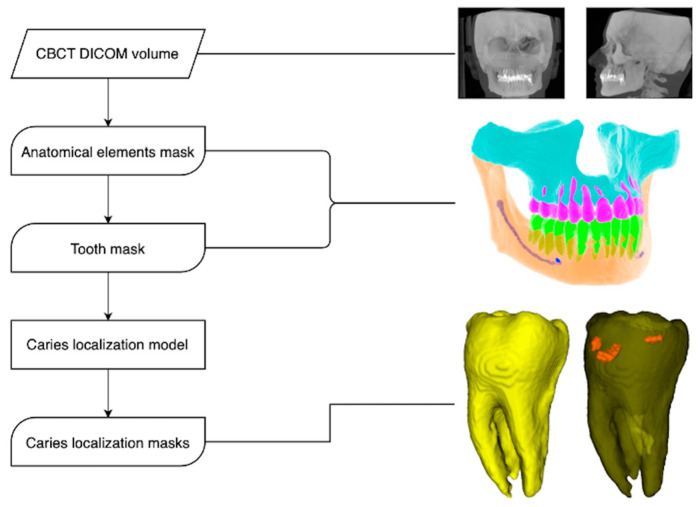
Diagnocat system model pipeline.

The Diagnocat system generates a radiological report based on a pipeline of multiple pre-trained fully CNN and algorithmic slice extraction. A radiological report includes a panoramic reformat of a CBCT and a section with slices and evaluations for each tooth (Figure 3). Predictions crucial for signs of caries evaluation include only voxel-perfect segmentations of teeth, although segmentations of different anatomy elements are also used for other evaluations (e.g., orthodontic aid).

**Figure 4:**
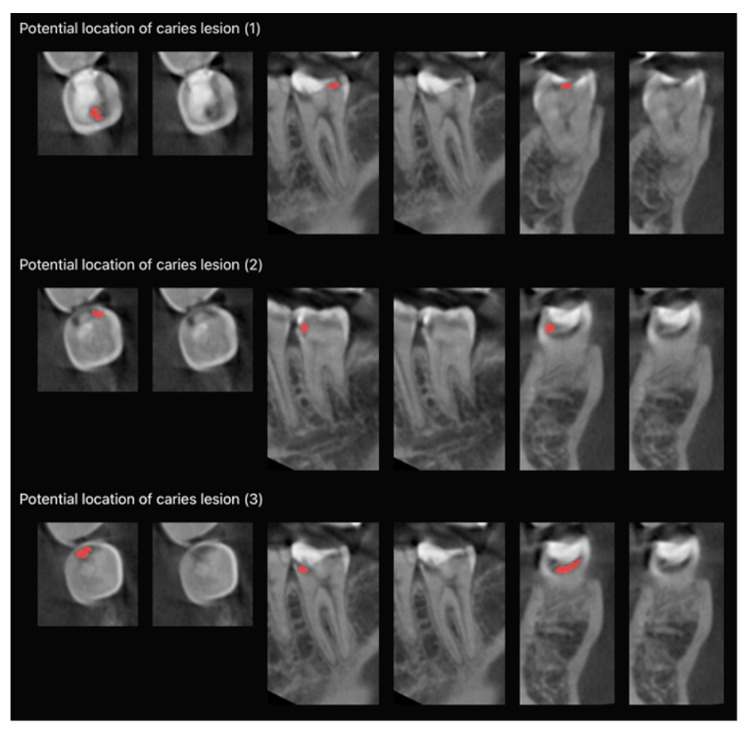
Extended slice section of aided evaluations (Diagnocat) showing separate predicted caries lesion masks (red) in axial, mesiodistal, and buccolingual views of tooth 37.

The tooth volume was cropped from a CBCT using a boundary box of a tooth segmentation mask extended by 3 mm from each side of the box. It was rescaled to have isotropic voxels and a 0.25 mm voxel size and resized to a fixed shape of 96 × 64 × 64. The tooth volume was fed to a caries localization model, and the model prediction was rescaled to the original voxel size and resized to an initial tooth volume shape. During the first step of post-processing of the caries localization model, the predicted caries lesion mask was labeled by connected components, resulting in a set of separate predicted lesions situated inside the tooth. Lesions with a volume less than 0.3 cm^3^ were ignored. The magnitude of volume threshold was derived from the training dataset lesion volume distribution. During the second step, predicted probability from the classification head of the model was rescaled in a way that a probability value of 0.5 corresponded to the maximum score based on sensitivity and specificity. The last step of post-processing the caries localization model was an intersection of a segmentation mask of the tooth of interest with a predicted caries lesion mask. This step was used to eliminate from final prediction caries lesion predictions situated inside neighboring teeth. The intersection was conducted with a morphological operation of binary dilation of a tooth segmentation mask and a multiplication of boolean masks of a tooth and caries lesions. The final prediction masks were used as visualizations of caries lesion locations via imposition of lesion masks on the tooth volume in axial, mesiodistal, and frontal views (Figure 4).

### 2.2. The Architecture of the Deep CNNs

The Diagnocat system exploits a set of pre-trained semantic segmentation networks based on internally modified fully convolutional 3D U-Net architecture from Isensee et al. [37] to obtain voxel-perfect segmentation masks of teeth, caries lesions, and anatomical elements. As well as the original U-Net, the modified architecture consists of a contraction path (the encoder) that encodes abstract representations of the input, followed by a symmetric expanding path (the decoder) that takes into consideration these features with high dimensional feature representations to precisely localize regions of interest. Blocks of the encoder were connected by 3 × 3 × 3 convolutions with stride 2 to reduce the resolution of the feature maps. Nearest neighbor interpolation was used to up-sample the low-resolution feature maps. Blocks of the decoder consisted of 3 × 3 × 3 convolution followed by 1 × 1 × 1 convolution which halves the number of feature maps. Features at each up-sampling level were concatenated with the features from the corresponding level of the encoder. Additionally, in the localization pathway, we integrated segmentation layers at different levels of the network and combined them via element-wise summation to form the final network output.

Additive attention gates were used at each up-sampling level to highlight salient image regions and preserve only the activations relevant to the main task. In such a gate, a single scalar attention coefficient (in range from 0 to 1) was obtained for each pixel vector which corresponds to the number of feature maps at the current layer of the model. Finally, after the last step of the up-sampling pathway, the classification block was added to predict the probability of the input being pathological in conventional classification fashion.

Leaky ReLU non-linearities were used as an activation function throughout the architecture. Additionally, traditional batch normalization was replaced with instance normalization due to small batch sizes of 3D volumes.

All networks were not initialized with any pre-trained weights and were trained from scratch. The combination of Jaccard loss and cross-entropy loss was utilized for segmentation tasks. The anatomical elements were labeled separately, while all signs of caries were labeled as a single class and further assigned to specific teeth.

### 2.3. Statistical Analysis

The results of the observers on the five-point confidence scale were transformed into binary categories (score 1, 2, and 3 as ‘absence of caries’, score 4 and 5 as ‘presence of caries’), and the ground truth was determined by calculating the consensus of the observers. After cleaning the data, in case three observers scored the same, the result was considered ground truth, and conflicting surfaces were identified in online sessions under the supervision of senior dentomaxillofacial radiologists.

After a two-month-long time interval, 50 randomly chosen samples were evaluated again to calculate the intra-observer agreement for both aided and unaided evaluations. Consistency between the aided and the unaided results of the observers were evaluated by kappa statistics (95% Cl) for the assessment of intra- and inter-observer agreement. In addition, Fleiss kappa was used to demonstrate the agreement among all observers in their aided and unaided evaluations, regardless of the ground truth. Consistency between the binary results and the ground truth was analyzed with sensitivity, specificity, accuracy, and kappa statistics. Sensitivity, specificity, and accuracy values were calculated using Equation (1):

Sensitivity = TP⁄(TP + FN)
Specificity = TN⁄(TN + FP)
Accuracy = (TP + TN)⁄(TP + FP + TN + FP)(1)

(TP: True positive, FP: False positive, TN: True negative, FP: False positive)

Statistics were calculated using SPSS (Version 25). A *p*-value of less than 0.05 was determined as the threshold for statistical significance.

## 3. Results

The kappa and weighted kappa coefficients are interpreted as described by Viera et al. [38] in Table 1.

Intra-observer agreements of each observer are demonstrated by kappa coefficients in Table 2. For Cohen’s kappa coefficient values, Observer 3’s repeatability for the five-point scale scoring increased from substantial agreement to almost perfect agreement with software support.

Consistency between the observer’s aided and unaided evaluations is shown in Table 3. For binary results, Cohen’s kappa coefficients were found to be almost perfect agreement, substantial agreement, and moderate agreement for Observer 1, Observer 2, and Observer 3, respectively.

The distribution of absence or presence of caries in binary scores is demonstrated in Table 4. The ratio of absence/presence of caries in ground truth was found to be approximately 2.32.

**Figure 5:**
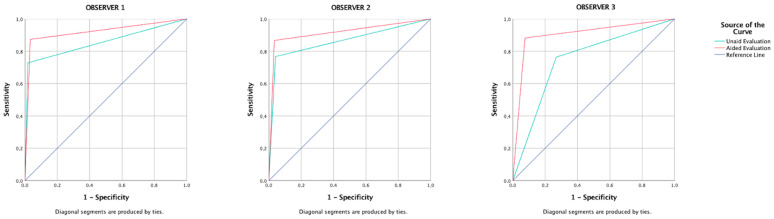
The ROC curves of aided and unaided evaluation of each observer.

The areas under the ROC curve of Observers 1, 2, and 3 were found to be 0.855, 0.863, and 0.747 for unaided and 0.920, 0.917, and 0.903 for aided (Figure 5) evaluations, respectively.

The general consensus among all observers in binary scores is shown in Table 5. Agreement among the three observers for the presence of caries changed from substantial agreement (Fleiss Kappa: 0.612) to almost perfect agreement (Fleiss Kappa: 0.829) in the aided results. Overall agreement changed from moderate agreement (Fleiss Kappa: 0.443) to substantial agreement (Fleiss Kappa: 0.757) in the binary results.

The general consensus among all observers on the five-point confidence scale is shown in Table 6. Overall agreement changed from fair agreement (Fleiss Kappa: 0.325) to moderate agreement (Fleiss Kappa: 0.468) on the five-point confidence scale.

The accuracy of the observers’ aided and unaided responses in determining the presence or absence of caries according to the ground truth along with kappa coefficients is shown in Table 7. The difference in accuracy between aided and unaided responses of the Observer 3 were found to be the highest, while Observer 1 achieved the best accuracy (0.939) in the aided results.

The agreement between the observers is shown in Table 8. The highest agreement was found between the aided results of Observer 1 and Observer 2, with substantial to almost perfect agreement for different types of kappa coefficients (five-point confidence scale quadratic results, weighted kappa: 0.859 and kappa: 0.664; binary results, kappa: 0.810).

## 4. Discussion

CBCT has become a very important radiographic technique in dentistry. The use of CBCT in dental procedures has gained popularity in recent years due to its low cost, fast image production rate, and lower radiation dose in comparison to medical CT [39]. However, CBCT machines are operated at milliamperes that are roughly one order of magnitude below the medical CT machines. Noise is defined as an unwanted disturbance of a signal that tends to obscure the signal’s information. Despite the reduction in the radiation dose, a high noise level or lower signal-to-noise ratio is expected in CBCT images. Noise reduces contrast resolution and affects the ability to segment low-density tissues effectively [40,41]. Artifact is any distortion or error in the image that is unrelated to the subject. Image artifacts are one of the drawbacks of the clinical use of CBCT. Artifacts may obscure or simulate the pathology of the head and neck region, including dental caries [39,41].

Scatter is caused by those photons that are diffracted from their original path after interaction with matter. The scattered photons are captured by the sensor and simply added to the primary intensity. The geometry of the detector is an important factor for this image-degrading effect of scattered radiation; as the sensor gets larger, the probability of catching a scattered photon is raised. Scatters reduce soft-tissue contrast and affect the density of all tissues [40]. The streak artifacts caused by scatter are very similar to those of beam hardening [40,41]. Beam hardening is one of the most common sources of artifacts. As the beam passes through the object, a highly absorbing material in the object, such as metal, can function as a filter to absorb the lower energetic photons more rapidly than the higher energetic photons. Hence, the beam spectrum becomes rich in high-energy photons and the mean energy increases. When the spectrum of the captured beam contains relatively more higher energetic photons than the emitted ray, the beam becomes ‘hardened’ and an artifact is induced, resulting in dark streaks [39,40]. Artifacts are related to several factors such as the object, material type, FOV, imaging device, and parameters [42,43]. The effectiveness of metal artifact reduction algorithm has been investigated by several authors [44,45,46]. Xie et al. proposed a deep CNN to reduce scatter artifacts for CBCT in an image-guided radiation therapy system [47].

Several authors investigated the potential of using CBCT instead of plain radiographs in detecting dental caries. Studies on this issue have reported varying results, perhaps due to differences in methodology. Young et al. evaluated the CBCT images in detecting proximal and occlusal caries by mounting 146 non-restored extracted human teeth in plaster. Caries lesions are categorized according to location and depth, and practicing dentists are found to be more successful in CBCT images with the average sensitivity of 0.61 when compared to plain radiographs but with not occlusal caries [48]. Kayipmaz et al. investigated the use of CBCT in detecting occlusal and approximal caries using 72 extracted human teeth. In their study, CBCT was reported to be superior in detecting not the approximal but the occlusal caries, when compared to plain radiographs [49]. Krzyzostaniak et al. conducted a study using 135 extracted human posterior teeth, and the accuracy of detecting non-cavitated proximal caries with CBCT unit was reported to be (0.629) inferior to other intra-oral radiography techniques. However, the CBCT system is reported to be slightly better for detecting occlusal carious lesions [50].

Unlike researchers that included occlusal caries, some studies excluded this location, as we have, and focused on approximal caries detection. Zhang ZL et al. evaluated 39 non-cavitated and unrestored human permanent teeth for approximal caries. The mean ROC values for two different CBCT devices were reported to be 0.528 and 0.525 (*p* = 0.763). The performance of CBCT was reported to be a little better than chance when compared to plain radiography [51]. Valizadeh et al. embedded 84 extracted human teeth in blocks, and the area under the ROC curve, sensitivity, specificity, accuracy, and positive and negative predictive values of CBCT images were reported to be 0.568, 0.835, 0.637, 0.714, 0.598, and 0.856, respectively. Afterall, CBCT images did not enhance the detection of proximal caries in comparison with plain radiography [52]. Wenzel et al. mounted 257 non-filled human teeth in plaster to be evaluated and found that CBCT was more accurate than intra-oral radiography in detecting approximal caries [53].

Several studies are performed with the motivation that the artifacts caused by restorative materials may affect the diagnosis of caries. Charuakkra et al. compared CBCT and bitewing radiographs in detecting secondary caries using 120 cavity slots with different restorative materials. The mean ROC values for the CBCT system were reported to be 0.995, and 0.978, making CBCT superior to bitewing radiographs [54]. Melo et al. evaluated the use of CBCT in detecting recurrent caries-like lesions by creating artificial caries lesions under restorative materials. In their study, CBCT and intra-oral radiography were found to be similar in detecting demineralization under restorations [55]. In addition, not all CBCT machines are duplicates due to the adoption of different production technologies. Considering that differences in production technology may affect the diagnosis of dental caries, Qu et al. investigated the effect of two different detector types employed in five CBCT systems on the diagnostic accuracy of approximal carious lesions by evaluating 78 approximal surfaces. According to the results of this study, the differences between five different CBCT devices and two different detector types were not found to be statistically significant [56].

In this study, the areas under ROC curves (0.747–0.863 for unaided, and 0.903–0.920 for the aided evaluations) were found to be better than those in Zhang ZL et al. (0.525–0.528) and Valizadeh et al. (0.568) and close to the Charuakkra et al. (0.978–0.995) [51,52,54]. Sensitivities (0.729–0.767 for unaided, 0.874–0.882 for aided evaluations) were found to be better than the research of Young et al. (0.61) and similar to the study of Valizadeh et al. (0.835) [48,52]. Higher coefficients in Table 2 can provide evidence to the higher repeatability of observers for evaluations without the CDSS support (Sante DICOM Viewer Pro) and with the software support (Diagnocat), separately. Repeatability values for all observers were found to be almost perfect, except for one variable. Unaided results of Observer 3 on the five-point scale evaluation were found to be in substantial agreement (0735) with Cohen’s kappa coefficient; however, the same observer reached almost perfect agreement (0.911) in the observations made with DiagnoCat system. The results of Table 3 may represent the magnitude of the decision changes before and after using the Diagnocat system, inversely. Kappa coefficients of Cohen’s and weighted were calculated in the range of fair agreement (0.438) and almost perfect agreement (0.816) for Observer 1, fair agreement (0.349) and substantial agreement (0.713) for Observer 2, and fair agreement (0.308) and substantial agreement (0.607) for Observer 3. Accordingly, the decision changes made with the use of the Diagnocat system were found to be minimal in Observer 1, while the magnitude of the decision changes in Observer 3 was found to be slightly greater than in Observer 2. According to Table 7, in unaided evaluations, Observer 1 achieved the lowest sensitivity (0.729) and highest specificity (0.982). While sensitivity values were calculated similarly for Observer 2 (0.767) and Observer 3 (0.764), the lowest specificity value was found in Observer 3 (0.731). In aided evaluations, sensitivity values were increased to 0.874, 0.867, and 0.882 for Observer 1, Observer 2, and Observer 3, respectively. Specificity values were improved in both Observer 2 (0.966) and Observer 3 (0.924), while there was some loss in Observer 1 (0.967). Afterall, accuracies of all observers were improved when using the Diagnocat system. Kappa coefficients for Observer 1 were changed from substantial agreement (0.759) to almost perfect agreement (0.852) for Observer 2, they were changed from substantial agreement (0.756) to almost perfect agreement (0.846); and for Observer 3, they were changed from moderate agreement (0.446) to substantial agreement (0.793) before and after using the Diagnocat system. According to the results, it can be thought that Observer 1 was more cautious in caries scoring than Observer 3. This difference may be due to the difference in approach in distinguishing artifacts from caries findings. The results of our research show that the impact of the Diagnocat system on clinicians’ decisions varies in magnitude and nature. Based on the findings, it can be suggested that Observer 3 is the one most affected by software support. The general consensus was improved from moderate agreement (0.443) to substantial agreement (0.757) using the software (Table 5), and the pair-wise agreements were improved (Table 8). While the findings of our research show that there is a general improvement in the evaluations made with Diagnocat, the slight decrease in the specificity of Observer 1 reminds us that these systems are not mistake-free and are auxiliary tools, and the importance of the final decision remains with the clinician.

Cardoso et al. defined gold standard data or methods as something that has already been checked (histologically, microscopically, chemically, etc.) and presents the best accuracy (sensitivity and specificity). Ground truth was reported as data and/or methods related to a consensus or more reliable values/aspects that can be used as references but were not or cannot be checked [57]. Experiments with extracted teeth allow for histopathological evaluation, and the lack of histopathological inspection can be considered as the limitations of this study. In our study, not the golden standard but ground truth was obtained and not for caries diagnosis but for radiographic caries detection. To address this point, a consensus was obtained among observers, similar to previous studies [58,59,60,61]. Thus, by using real patient images, there was no need to simulate conditions such as artifacts in translating the experimental results to clinical environment. Artifacts seen in CBCT imaging, especially those associated with metallic restorations, may affect the effectiveness of caries detection [32]. The lack of a distinction for restorations in our study can be considered as a limitation. In the meantime, samples with gross artifacts were not included in our study, and we aimed to overcome this situation by keeping the number of samples surplus. Thus, we aimed to reflect a realistic clinical situation by avoiding the bias caused by the distinction between images with and without artifacts. Evaluating the effect of artifacts due to restorations may be the subject of future studies. Caries identification by CBCT is a controversial topic and beyond the scope of this study. The developed system analyzes the volumetric data already saved and presents the dental caries signs to the operator. Further clinical review and final decision rests with the clinician. We suggest that machine learning tools, such as the system in our study, may be useful in detecting secondary findings, rather than for the primary imaging purpose, for better diagnosis and treatment planning.

## 5. Conclusions

In this study, radiographic evaluations performed by three observers were found to be more compatible and accurate with the aid of the AI system, when compared to the evaluations without the AI system, in detecting dental caries on CBCT images. Our study does not recommend justifying the use of CBCT imaging for caries diagnosis but suggests that once the volumetric data are acquired, machine learning tools can be helpful in detecting the caries signs. As technology advances, the integration of similar tools into the digital radiology workflow can assist clinicians in evaluating radiographic data.

## Figures and Tables

**Table 1:** Interpretation of Kappa statistics [38].

Kappa	Agreement
<0	Less than change agreement
0.01–0.20	Slight agreement
0.21–0.40	Fair agreement
0.41–0.60	Moderate agreement
0.61–0.80	Substantial agreement
0.81–0.99	Almost perfect agreement
1	Perfect agreement

**Table 2:** Intra-observer agreement for unaided and aided evaluations.

	Cohen’s Kappa				Weighted Kappa			
	Five-Point Scale		Binary Scale		Five-Point Scale		Binary Scale	
	Unaided	Aided	Unaided	Aided	Unaided	Aided	Unaided	Aided
Observer 1	0.820	0.903	0.926	0.939	0.938	0.958	0.926	0.939
Observer 2	0.903	0.923	0.932	0.945	0.962	0.968	0.932	0.945
Observer 3	0.735	0.911	0.865	0.939	0.849	0.937	0.865	0.939

**Table 3:** Consistency between each observer’s aided and unaided results.

Score	Kappa	Observer 1	Observer 2	Observer 3
Multiple	Weighted	0.749	0.713	0.607
	Cohen’s	0.438	0.349	0.308
Binary	Cohen’s	0.816	0.683	0.410

**Table 4:** Distribution of absence or presence of caries for binary scores.

*n*		Absence	Presence
Observer 1	Unaided	15,302	4634
	Aided	14,225	5711
Observer 2	Unaided	14,756	5180
	Aided	14,253	5683
Observer 3	Unaided	11,604	8332
	Aided	13,584	6352
Ground Truth		13,928	6008

**Table 5:** General consensus among all observers in binary scores.

Fleiss Kappa Coefficient	Binary	
Tooth Condition	Unaided	Aided
1. Absence of caries	0.831	0.928
2. Presence of caries	0.612	0.829
Overall Consensus	0.443	0.757

**Table 6:** General consensus among all observers in multiple scores.

Fleiss Kappa Coefficient	Multiple	
Tooth Condition	Unaided	Aided
1. Definitely not	0.534	0.581
2. Probably not	0.494	0.628
3. Not sure	0.420	0.330
4. Probably yes	0.305	0.709
5. Definitely yes	0.561	0.668
Overall Consensus	0.325	0.468

**Table 7:** The sensitivity and specificity of unaided and aided evaluations of each observer.

		TP	TN	FP	FN	Sensitivity	Specificity	Accuracy	Kappa
Observer 1	Unaided	4377	13,671	257	1631	0.729	0.982	0.905	0.759
	Aided	5248	13,465	463	760	0.874	0.967	0.939	0.852
Observer 2	Unaided	4609	13,357	571	1399	0.767	0.959	0.901	0.756
	Aided	5210	13,455	473	798	0.867	0.966	0.936	0.846
Observer 3	Unaided	4588	10,184	3744	1420	0.764	0.731	0.741	0.446
	Aided	5299	12,875	1053	709	0.882	0.924	0.912	0.793

FN: False negative, FP: False positive, TN: True negative, TP: True positive.

**Table 8:** Pairwise agreement among observers.

Observer			Five-Point Confidence Scale				Binary Score	
			Weighted Kappa		Cohen’s Kappa			
1	2	3	Unaided	Aided	Unaided	Aided	Unaided	Aided
X	X		0.557	0.859	0.332	0.664	0.583	0.810
X		X	0.406	0.578	0.355	0.420	0.388	0.740
	X	X	0.435	0.574	0.317	0.373	0.408	0.724

## Data Availability

The data presented in this study are available on request from the corresponding author.
